# Maternal puerperal infection caused by *Parabacteroides goldsteinii*: a case report

**DOI:** 10.3389/fmed.2024.1450931

**Published:** 2024-08-09

**Authors:** Liwei Peng, Xiaomin Chen, Zhenhui Wang, Lu Yi, Zhengjiang Jin

**Affiliations:** ^1^Department of Clinical Laboratory, Maternal and Child Health Hospital of Hubei Province, Tongji Medical College, Huazhong University of Science and Technology, Wuhan, China; ^2^Department of Disinfection and Pest Control, Wuhan Center for Disease Control & Prevention, Wuhan, Hubei, China

**Keywords:** *Parabacteroides goldsteinii*, sepsis, postpartum, infection, drug-resistance

## Abstract

**Background:**

*Parabacteroides goldsteinii,* a member of the *Parabacteroides genus*, was initially discovered in the feces and abdominal tissue of patients with appendicitis, peritonitis, and abdominal abscesses. In recent years, *P. goldsteinii* has been widely regarded as a gut probiotic, and human infections have been extremely rare. In 2010, *P. goldsteinii* was first isolated from the blood culture of a patient with abdominal infection, confirming its ability to cause bacteremia. In this study, we report a rare case of puerperal infection with septic shock caused by *P. goldsteinii* infection in a pregnant woman.

**Case presentation:**

A 31-year-old female experienced perineal lacerations, cervical lacerations, and postpartum hemorrhage during childbirth. Nine days postpartum, the patient developed septic shock, and *P. goldsteinii* infection was identified through blood culture and mass spectrometry. We administered broad-spectrum antibiotics, including meropenem/nalidixic acid and piperacillin tazobactam, intravenously, but the antimicrobial effect was not satisfactory. Upon ultrasound examination, we identified a focus of infection in the patient’s uterus. Subsequently, uterine curettage was performed, followed by uterine cavity irrigation with metronidazole and intramuscular injection of gentamicin and dexamethasone. Following treatment, the patient’s physiological parameters gradually returned to normal, and she was discharged 30 days after admission.

**Conclusion:**

*Parabacteroides goldsteinii* bacteraemia is extremely rare, and clinically, the postinfection toxicity of this bacterium appears to be significant. In this report, we review the research history of *P. goldsteinii* and relevant infection cases, aiming to enhance awareness among clinical practitioners, particularly obstetricians and gynecologists, regarding *P. goldsteinii* bloodstream infections, facilitating early diagnosis and timely treatment.

## Background

*Parabacteroides goldsteinii* is an obligate anaerobic gram-negative rod-shaped bacterium that measures 0.9 to 1.5 m by 1.2 to 10 m and grows as gray, circular, convex, opaque colonies with a diameter of 1 to 2 millimeters on Brucella blood agar after 48 h of incubation. It was initially isolated from the feces and abdominal tissue of three patients with appendicitis, three patients with peritonitis, and three patients with abdominal abscesses and was named *Bacteroides goldsteinii* (1). Subsequently, three *Bacteroides* species, *Bacteroides distasonis*, *Bacteroides goldsteinii* and *Bacteroides merdae*, were reclassified as *Parabacteroides* (2). Previous reports have described cases of abdominal perforation in patients infected with *P. goldsteinii* and bacteremia, but there are no reports of cases in postpartum women infected with the bacterium. Consequently, we report the first case of a patient with postpartum septic shock and bacteremia with *P. goldsteinii* isolated from blood culture, indicating that *Parabacteroides goldsteinii* is highly pathogenic in the puerperium.

## Case presentation

The subject of our report is a 31-year-old female who was admitted to the Maternal and Child Health Hospital of Hubei Province for delivery on February 3, 2024. Upon admission, her physical examination results were largely normal: T 36.6°C, P100 beats/min, R20 breaths/min, and BP 105/68 mmHg. Her fundal height was 38.0 cm, her abdominal circumference was 104.0 cm, her fetal heart rate was 138 beats/min, there were no uterine contractions, her cervix was closed, her membranes were intact, her pelvic examination was normal, and no significant past medical history was reported. Two days after admission, on February 5, 2024, she delivered a live male infant via spontaneous left occiput anterior (LOA) vaginal delivery, with a birth weight of 4,050 g. Due to fetal macrosomia, the delivery process resulted in second-degree lacerations of the perineum and cervix, with a postpartum hemorrhage of approximately 3,000 mL. To control bleeding, a Bakri balloon was immediately placed in the cervix and filled with 500.0 mL of normal saline. Additionally, vaginal suturing was performed. Despite suturing, the patient exhibited coagulation dysfunction and continued vaginal bleeding, necessitating further vaginal packing for hemostasis and bilateral uterine artery embolization. She received transfusions of 12 units of packed red blood cells, 9.5 units of cryoprecipitate, 1,400 mL of plasma, 4 g of fibrinogen, and 1 unit of platelets. Due to postpartum hemorrhage, coagulation dysfunction, and hemorrhagic shock, the patient was transferred to the intensive care unit (ICU) for further management.

During her week-long ICU stay, the patient received intravenous cefoperazone/sulbactam (Sulperazone). On the eighth day, laboratory tests, including complete blood count and high-sensitivity C-reactive protein (hs-CRP) levels, revealed elevated inflammatory markers: white blood cell count 10.84*10^9/L, hemoglobin 86 g/L, hs-CRP 14.45 mg/L, and serum amyloid A 95.36 mg/L. Given the ongoing epidemic of *Mycoplasma pneumoniae* during her hospitalization, immunological testing revealed positivity for IgM antibodies against *Mycoplasma pneumoniae*. Consequently, the patient was treated with oral azithromycin tablets (Zithromax, Pfizer) and oseltamivir phosphate capsules (Tamiflu, Shanghai). On the ninth day, 1 day after discontinuing intravenous cefoperazone/sulbactam, the patient developed fever with chills, with a temperature of 39.9°C, and was subjected to bedside resuscitation due to septic shock. Her neutrophil ratio reached 90.3%. Repeat laboratory tests on the tenth day revealed severe infection, likely septicemia. Blood cultures were obtained during the febrile period. Concurrently, antibiotic therapy was escalated to intravenous meropenem (1.0 g q8h) and linezolid (0.6 g Q12H), with the addition of potassium permanganate solution to the sitz baths. On February 17, the laboratory reported positive blood cultures for *Parabacteroides goldsteinii*. According to descriptions in “Fever: Sanford Guide to Antimicrobial Therapy,” no specific susceptibility testing standards are available for this anaerobic bacterium. Literature reports suggest that the first-line treatments for this bacterium are metronidazole or piperacillin/tazobactam, with second-line options including doripenem, ertapenem, imipenem, meropenem, and amoxicillin/clavulanic acid. Therefore, we adopted a treatment regimen comprising intravenous injection of piperacillin/tazobactam (4.5 g Q8H) combined with vaginal irrigation using metronidazole. On the twelfth day postpartum, the patient continued to have fever. Abdominal ultrasound revealed multiple hyperechoic areas within the uterine cavity measuring approximately 5.35.63.9 cm with indistinct borders, suggesting the presence of intrauterine infection foci. We hypothesized that *Parabacteroides goldsteinii* likely entered the bloodstream through these intrauterine infection foci, causing bacteremia. Therefore, we performed uterine curettage to remove the infected foci, followed by irrigation of the uterine cavity with metronidazole and intramuscular injection of gentamicin (2 mL) plus dexamethasone (1 mL). Postsurgery, there was a significant decrease in the patient’s blood count and inflammatory markers. Complete blood count and high-sensitivity C-reactive protein (hs-CRP) showed a white blood cell count of 9.0610^9/L, red blood cell count of 3.0010^12/L↓, hemoglobin of 90 g/L↓, platelet count of 45,310^9/L↑, neutrophil ratio of 82.5%↑, and hs-CRP of 87.18 mg/L↑. However, the patient’s CRP level continued to increase, so we increased the dosage of metronidazole to 0.5 g (q6h) while maintaining the original dose of piperacillin/tazobactam at 4.5 g (q8h) and administered a second dose of intramuscular gentamicin (2 mL) plus dexamethasone (1 mL). On the sixteenth day postpartum, the patient maintained a normal body temperature for two consecutive days, with significant improvement in infection indicators. Therefore, we reduced the dose of piperacillin/tazobactam to 4.5 g (q12h). After continuing treatment for two more days, the patient’s white blood cell count was 8.6910^9/L, and her hs-CRP was 7.53. Following a period of observation, the patient’s infection-related parameters essentially returned to normal, with body temperature remaining within the normal range, leading to discharge on February 25, 2024.

## Microbiological analysis and molecular examination

**Figure 1:**
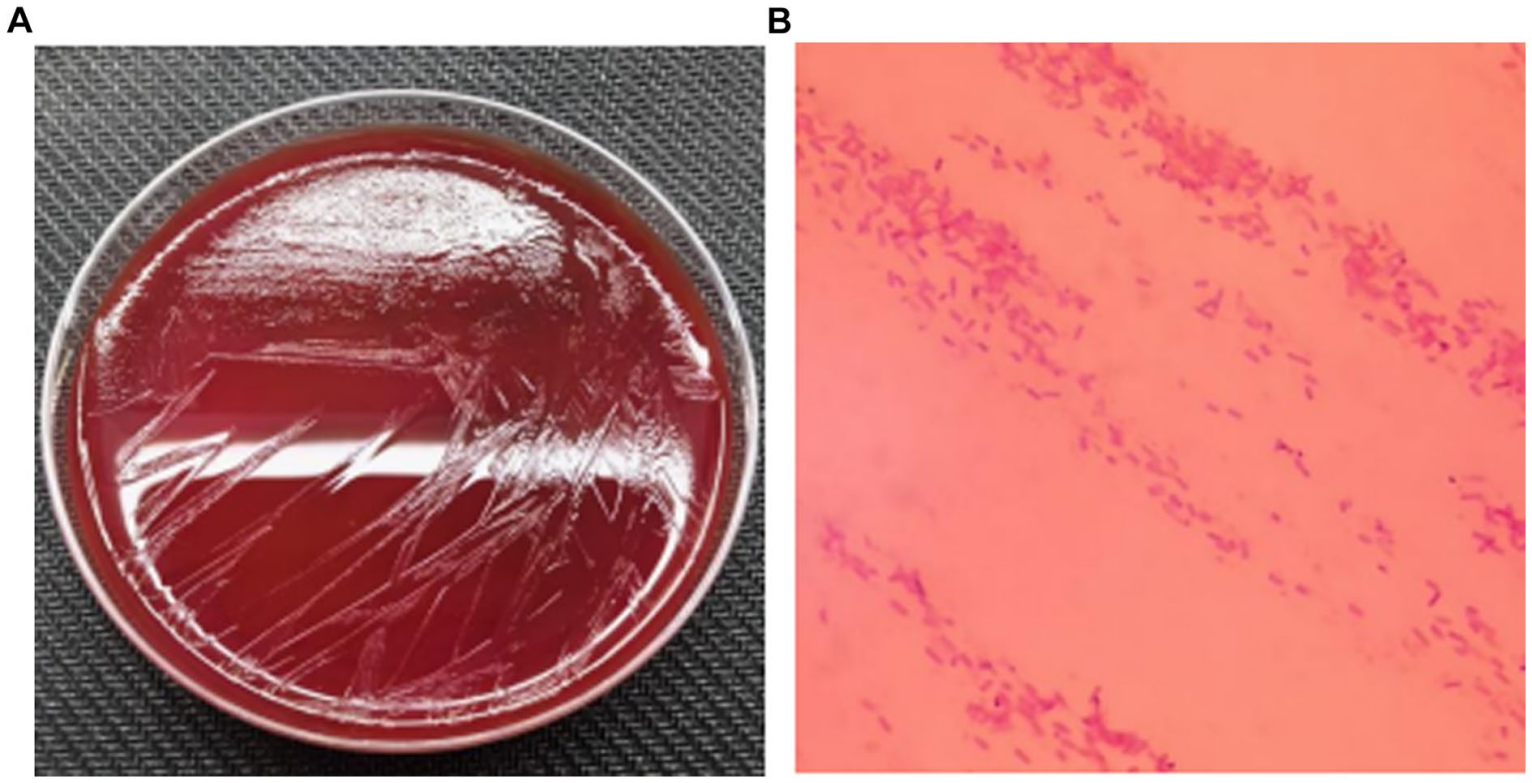
**(A)** Subcultured colony morphology on blood agar plate; **(B)** Gram staining from positive blood culture (*1000).

During the patient’s febrile period with chills, a set of blood cultures (both aerobic and anaerobic) was collected and sent to the microbiology laboratory for cultivation. After 39.5 h, the anaerobic bottle reported positive growth. The blood from the anaerobic bottle was subcultured onto Columbia blood agar plates and incubated under anaerobic conditions at 35°C for 24 h. Subsequently, small colonies with smooth edges, appearing gray–white and translucent, were observed on the plates (Figure 1A). Microscopic examination following Gram staining revealed gram-negative, short rod-shaped bacteria (Figure 1B). The identification of the bacterium *Parabacteroides goldsteinii* was performed using laser-assisted desorption/ionization time-of-flight (Microflex LT/SH, Bruker Daltonics). Then, we sequenced the whole genome of the bacterium and searched for the resulting sequence via the species Finder function of the Center for Genomic Epidemiology. The search results indicated that the bacterium was indeed *Parabacteroides goldsteinii*. Based on our genome sequencing results, the results are shown in Table 1, with 12 resistance loci in the genome against which it is likely to be resistant to sulfamethoxazole 12. Notably, an unknown beta-lactam was also encoded in the resistance gene.

**Table 1:** Analysis of drug resistance genes of *P. goldsteinii.*

Antimicrobial	Class	Match	Genetic background
Unknown beta-lactam	Beta-lactam	3	blaOXA-347 (blaOXA-347_ACWG01000053)
Sulfamethoxazole	Folate pathway antagonist	3	sul2 (sul2_AY034138)
Tetracycline	Tetracycline	3	tet(Q) (tet(Q)_Z21523), tet(X) (tet(X)_M37699)
Doxycycline	Tetracycline	3	tet(Q) (tet(Q)_Z21523), tet(X) (tet(X)_M37699)
Minocycline	Tetracycline	3	tet(Q) (tet(Q)_Z21523), tet(X) (tet(X)_M37699)
Lincomycin	Lincosamide	2	erm(F) (erm(F)_M17808)
Clindamycin	Lincosamide	2	erm(F) (erm(F)_M17808)
Erythromycin	Macrolide	2	ere(D) (ere(D)_KP265721), erm(F) (erm(F)_M17808)
Tigecycline	Tetracycline	2	tet(X) (tet(X)_M37699)
Quinupristin	Streptogramin b	2	erm(F) (erm(F)_M17808)
Pristinamycin ia	Streptogramin b	2	erm(F) (erm(F)_M17808)
Virginiamycin s	Streptogramin b	2	erm(F) (erm(F)_M17808)

## Discussion and conclusions

In recent years, *Parabacteroides goldsteinii* has been widely studied as a probiotic in the gut, and it is likely to have anti-inflammatory and antiobesity effects (3, 4). There have been few reports on the pathogenicity of *Parabacteroides goldsteinii*. In 2010, *Parabacteroides goldsteinii* was first isolated from a patient with bacteremia via blood culture (5), confirming that it is a human pathogen. Later, Cobo et al. also isolated *Parabacteroides goldsteinii* from the ascitic fluid and intestinal biopsy of a patient with lymphoma and abdominal infection (6). In both cases, the patients had peritonitis caused by intestinal perforation, suggesting that the gut is a possible source of infection. However, this is the first report of a pregnant woman who developed postpartum bacteremia due to *Parabacteroides goldsteinii,* which led to septic shock, and a single *Parabacteroides goldsteinii* was directly cultured from her blood. This finding has important clinical significance. Studies have shown that there is a significant change in the vaginal flora of postpartum women, with an increase in the proportion of anaerobic bacteria compared to before (7). Factors such as increased invasive procedures during delivery, episiotomy, vaginal lacerations, wounds in the vagina and uterus, and physiological changes in the immune system of postpartum women can increase the likelihood of postpartum anaerobic bacterial infections.

Song et al. used the agar dilution method to test the sensitivity of the six *Parabacteroides goldsteinii* strains they isolated. The results showed that the strains were sensitive to metronidazole (MIC, ≤2 μg/mL) and ertapenem (MIC, ≤1 μg/mL). There was some resistance to clindamycin (MIC, ≤8 μg/mL). All strains showed resistance to penicillin G (MIC, ≥32 μg/mL), cefotetan (MIC, ≤256 μg/mL), and vancomycin (MIC, ≤32 μg/mL). All strains were β-lactamase positive. Cobo et al. used the E-test to test amoxicillinclavulanic acid (8 mg/L), piperacillin-tazobactam (6 mg/L), clindamycin (> 256 mg/L), meropenem (0.5 mg/L), imipenem (3 mg/L), moxifloxacin (0.25 mg/L), and metronidazole (0.25 mg/L). Our patient received treatment with multiple antibiotics. After developing septic shock, she was continuously administered piperacillin-tazobactam and metronidazole for 7 days in combination with meropenem and linezolid; gentamicin and linezolid were used in combination therapy. Her inflammatory markers returned to normal, consistent with the literature’s reported drug sensitivity results.

The present case indicates that *P. goldsteinii* is highly pathogenic in the postpartum period in women, can cause bacteremia and septic shock, and can lead to serious consequences. The special physiological conditions of postpartum women greatly increase the probability of anaerobic bacterial infections. Song et al. also noted that *P. goldsteinii* is phenotypically similar to *P. merdae* (1), and the API ZYM, rapid ID 32A, and rapid ANA II systems often misidentify *P. goldsteinii* as *P. merdae*; this suggests that *P. goldsteinii* may be more frequently found in clinical specimens than previously thought, and clinicians, especially obstetricians, should pay attention to *P. goldsteinii* and other related anaerobic bacteria.

## Data availability statement

The original contributions presented in the study are included in the article/supplementary material, further inquiries can be directed to the corresponding author.

## Ethics statement

The studies involving humans were approved by the Ethics Committee of the Maternal and Child Health Hospital of Hubei Province. The studies were conducted in accordance with the local legislation and institutional requirements. Written informed consent for participation was not required from the participants or the participants’ legal guardians/next of kin in accordance with the national legislation and institutional requirements. Written informed consent was obtained from the individual(s) for the publication of any potentially identifiable images or data included in this article.

## Author contributions

LP: Data curation, Methodology, Writing – original draft, Writing – review & editing. XC: Data curation, Methodology, Writing – review & editing. ZW: Data curation, Formal analysis, Methodology, Writing – review & editing. LY: Data curation, Formal analysis, Methodology, Writing – review & editing. ZJ: Conceptualization, Investigation, Project administration, Writing – original draft, Writing – review & editing.
